# Increased Dysfunctional and Plastic Regulatory T Cells in Idiopathic Orbital Inflammation

**DOI:** 10.3389/fimmu.2021.634847

**Published:** 2021-05-03

**Authors:** Jingqiao Chen, Huijing Ye, Wei Xiao, Yuxiang Mao, Siming Ai, Rongxin Chen, Xiufen Lian, Lu Shi, Xing Wang, Shaowei Bi, Shenglan Yang, Xian Ji, Te Zhang, Huasheng Yang

**Affiliations:** State Key Laboratory of Ophthalmology, Zhongshan Ophthalmic Center, Sun Yat-sen University, Guangzhou, China

**Keywords:** regulatory T cell, dysfunction, plasticity, interleukin-33, idiopathic orbital inflammation, IgG4-related disease

## Abstract

**Background:**

Idiopathic orbital inflammation (IOI) is a disfiguring and vision-threatening fibroinflammatory disorder. The pathogenesis of IOI has not been elucidated. We sought to clarify the regulatory T cell (Treg) distribution and function in patients with IOI.

**Methods:**

The frequency, phenotype and function of Tregs were identified by multicolor flow cytometry and *in vitro* cell functional assays. Plasma and tissue samples were obtained to investigate cytokines, chemokines and their receptors of interest by relative real-time polymerase chain reaction (PCR) and Luminex assays.

**Results:**

Compared with healthy subjects, patients with IOI exhibited obvious increases of Tregs in peripheral blood and affected orbital tissues. Circulating Tregs from patients with IOI were significantly more polarized to a Th17-like phenotype with defective regulatory function, whereas orbit-derived Tregs were polarized to a Th2-like phenotype. Furthermore, ST2 expression levels in circulating Tregs and interleukin (IL)-33 mRNA levels in orbital tissues were decreased in IOI. IL-33 restored the suppressive function of Tregs, reduced interferon (IFN)-γ production by Tregs and decreased the activation of orbital fibroblasts (OFs) cocultured with Tregs in IOI.

**Conclusion:**

Increased Tregs with proinflammatory and profibrotic polarization were first identified in IOI, suggesting that Treg plasticity and heterogeneity plays an essential role in IOI pathogenesis. Additionally, our study identified a regulatory effect of IL-33 on inflammation and fibrosis in IOI. Reversing the plastic Tregs *via* IL-33 might be a potential option for IOI patients.

## Introduction

Idiopathic orbital inflammation (IOI) is a fibroinflammatory disease entity characterized by an enlarged orbital structure or mass (1). The clinical process ranges from inflammatory swelling to devastating sclerosis with blindness (2). Recently, IOI is divided into IgG4-related ophthalmic disease (IgG4-ROD) and non-IgG4-related IOI according to the diagnostic criteria for IgG4-related disease (IgG4-RD) (3). As little is known about the pathogenesis of IOI, there are currently no specific treatments, and recurrence rates are high.

The immune-mediated process of IOI has been postulated and is strongly supported by the observation that IOI responds rapidly to corticosteroid treatment (4). Pathologically, lesions in patients with IOI show varying degrees of fibrosis and infiltration of mixed inflammatory cells, including T helper (Th) cells and suppressive T cells (5). Th cells are counterbalanced by regulatory T cells (Tregs), the best characterized suppressive cell subset involved in maintaining immune homeostasis by limiting deleterious immune responses (6, 7). Extensive research into the contribution of Tregs in IgG4-RD led to consistent results indicating that IgG4-RD can be considered a modified Th2 response with increased Th2 cells and Tregs (8); however, nothing is known about the phenotype and function of these Tregs. Research has confirmed that disruption of Treg function facilitates the progression of inflammatory diseases (6). However, no comprehensive studies have demonstrated the role of Tregs in the progression of IOI.

A wealth of studies has shown that Tregs possess functional heterogeneity and plasticity (9). Based on chemokine receptor expression, plastic Tregs, which we refer to as Th-like Tregs, are divided into different subsets with migratory and functional properties that match the immune environment (10, 11). In certain disease settings, Tregs are reprogrammed toward a proinflammatory state (12) characterized by properties including the production of interferon (IFN)-γ, interleukin (IL)-17, or both (13, 14). Notably, IL-17-producing Tregs from patients with rheumatoid arthritis (RA) retain their suppressive function in peripheral blood but lose this function in RA synovial fluid (15), suggesting that Tregs may perform different roles in different locations. These interesting findings drove our research focus toward the plasticity of Tregs in IOI.

To better map these aspects of Tregs in patients with IOI and compare them with those of Tregs in healthy subjects, we performed the first comprehensive phenotypic and functional analysis of blood-derived and orbit-derived Tregs in patients with IOI.

## Materials and Methods

### Subjects

Patients with IOI were enrolled based on histopathologic diagnosis. Peripheral blood and affected orbital tissues from IOI patients were collected during biopsy or surgical debulking. Patients with a history of immunodeficiency or any autoinflammatory disease were excluded. Additionally, orbital inflammation activity was measured by the modified Werner grading system (score >3 was considered active) (16, 17). The individuals in the control group were age- and sex- matched and showed no symptoms of active inflammation. Control blood was obtained from healthy volunteers. Normal lacrimal glands were obtained from patients with lacrimal gland prolapse, and normal orbital fat was obtained from subjects who underwent blepharoplasty or eye enucleation. The basic characteristics of the patients with IOI and control subjects are summarized in **Table S1**. This work adhered to the tenets of the Declaration of Helsinki and was approved by the Institutional Review Board of Zhongshan Ophthalmic Center, Sun Yat-sen University. All patients and healthy donors enrolled in the study provided written informed consent.

### Cell Culture

Orbital fibroblasts (OFs) were cultured from fresh orbital samples (orbital tumors and normal orbital fat) after biopsy or surgical excision. Samples were minced, put in petri dishes and cultured in Dulbecco’s modified Eagle’s medium (DMEM) (Gibco, Waltham, MA, USA) supplemented with 10% fetal bovine serum (FBS) (Gibco), streptomycin (100 mg/mL) (Gibco) and penicillin (100 mg/mL) (Gibco) for 2-3 weeks at 37°C. All experiments were performed with OFs up to passage 6. OFs (5×10^5^ cells) were stimulated with different concentrations (0, 1, 10, 20, 50, 100 ng/ml) of human (rh)IL-33 (R&D Systems, Minneapolis, MN, USA) for 24, 48, and 72 hours. For coculture experiments, sorted Tregs (1×10^5^ cells) from IOI patients were resuspended in Iscove’s modified Dulbecco’s medium (IMDM) (Gibco) and seeded in plates on pre-plated fibroblasts (1×10^5^ cells) for 72 hours.

### Histologic Staining

Orbital samples were formalin-fixed, paraffin-embedded and sectioned. Slides were deparaffinized and rehydrated and were subsequently subjected to Alcian blue/periodic acid-Schiff (AB-PAS) staining (Servicebio, Wuhan, Hubei, China) and Masson trichrome staining (Servicebio). Sections were examined with a microscope (Nikon, Tokyo, Japan).

### Cell Separation

Peripheral blood mononuclear cells (PBMCs) were isolated by Ficoll-Paque density gradient centrifugation (GE Healthcare, Marlborough, MA, USA). Lymphocytes from orbital tumors, normal orbital fat or lacrimal glands were isolated by mincing fresh specimens into 1-mm^3^ pieces and performing enzymatic digestion using a Tumor Dissociation Kit (Miltenyi Biotec, Bergisch-Gladbach, Germany) for 30-60 minutes at 37°C, according to the manufacturer’s instructions. The dissociated cells were passed through a 40 μm filter and centrifuged. Then, the pelleted cells were suspended in red blood cell lysis buffer (Miltenyi Biotec) and incubated on ice for 2 minutes. The suspension was subsequently centrifuged and washed twice prior to staining.

### Absolute Count of Tregs

BD Trucount™ Tubes (BD Biosciences, Franklin Lakes, NJ, USA) were used to determine the absolute counts of Tregs. Well-mixed anti-human CD3 (SK7, BD Biosciences), anti-human CD4 (RPA-T4, BD Biosciences), anti-human CD25 (M-A251, BD Biosciences), and anti-human CD127 (HIL-7R-M21, BD Biosciences) antibodies were added above the stainless steel before pipetting anticoagulated whole blood onto tubes. Tubes were vortexed and incubated. Then lysing solution was added to the tubes and mixed. The samples were incubated before to be analyzed.

### Flow Cytometry

Single-cell suspensions were initially incubated with an Fc receptor blocking antibody (Biolegend, San Diego, CA, USA) and stained with the following antibodies for surface analysis: anti-human CD3 (SK7, BD Biosciences), anti-human CD4 (RPA-T4, BD Biosciences), anti-human CD25 (M-A251, BD Biosciences), anti-human CD127 (HIL-7R-M21, BD Biosciences), anti-human CXCR3 (1C6, BD Biosciences), anti-human CCR4 (L291H4, Biolegend), and anti-human CCR6 (G034E3, Biolegend). PBMCs were stained with anti-CD3 (SK7, BD Biosciences), anti-CD4 (RPA-T4, BD Biosciences), anti-CD25 (M-A251, BD Biosciences) prior to fixation, permeabilization and staining for Foxp3 (259D, Biolegend). For analysis of ST2^+^ Tregs, PBMCs were stained for CD4 and ST2 (RMST2-33, Invitrogen, Carlsbad, CA, USA) prior to staining for Foxp3. For analysis of α-smooth muscle actin (α-SMA)^+^ OFs after coculture with Tregs, OFs were fixed and permeabilized after harvest and stained with an anti-α-SMA antibody (1A4/asm-1, Novus Biologics, Minneapolis, USA).

### Treg Functional Assay

Naïve T cells (responder cells) were sorted using a Naïve CD4+ T Cell Isolation Kit (Miltenyi Biotec) according to the manuscript, and labeled with carboxyfluorescein diacetate succinimidyl ester (CFSE) (Invitrogen). FACSAria Fusion (BD Bioscience) was used for Treg sorting. Sorted Tregs (1×10^5^ cells) were cocultured with sorted naïve T cells (1:2) for 5 days. The proliferation of naïve T cells was measured by flow cytometry. In addition, the cytokine measurement was performed after cocultured FACS-sorted Conventional CD4+ T cells (Tconvs) with or without Tregs (1:1) purified from PBMCs for 3 days. For the final 4-6 hours, cells were stimulated with phorbol 12-myristate 13-acetate (PMA) (50 ng/mL) (Sigma-Aldrich, St. Louis, MO, USA), ionomycin (1 µg/mL) (Sigma-Aldrich) and brefeldin A (5 µg/mL) (Biolegend) before harvest to determine the percentages of tumor necrosis factor (TNF)-α^+^CD4^+^, IFN-γ^+^CD4^+^, IL-4^+^CD4^+^, and IL-17A^+^CD4^+^ T cells. T cells were stained with anti-CD4 (RPA-T4, BD Biosciences) prior to fixation, permeabilization and staining for TNF-α (Mab11, Biolegend), IFN-γ (4S. B3, BD Biosciences), IL-4 (8D4-8, BD Biosciences), and IL-17A (N49-653, BD Biosciences). In some experiments of CFSE assay and cytokine measurement, Tregs were stimulated with recombinant human (rh)IL-33 (20 ng/mL) (R&D Systems) and human anti-ST2 mAb (R&D Systems), which competitively bound to the IL-33 receptor (ST2). Cells were cocultured in RPMI-1640 medium (Gibco) supplemented with 10% FBS (Gibco) in the presence of anti-CD3 (1ug/mL) (R&D Systems), anti-CD28 (1 µg/mL) (R&D Systems) and IL-2 (10 ng/mL) (PeproTech, Rocky Hill, NJ, USA).

### Quantitative Real-Time Reverse Transcription Polymerase Chain Reaction (qRT-PCR)

Total RNA from orbit explants was extracted with TRIzol (Invitrogen), and reverse transcription was then performed using a PrimeScript RT Reagent Kit (Takara). qRT-PCR was performed with SYBR Premix Ex Taq (Takara) according to the manufacturer’s instructions. GAPDH was used as the internal control, and the relative expression levels were calculated by using the 2^-ΔΔCT^ method with normalization to GAPDH. The primers used in this study were as follows: *CXCL9* (forward), 5’-GGAAGCAGCCAAGTCGGTTA-3’; and (reverse) 5’-AGGAGGTTTCCACATCCTGC-3’; *CXCL10* (forward), 5’-CTGAGCCTACAGCAGAGGAAC-3’; and (reverse), 5’-GATGCAGGTACAGCGTACAGT-3’; *CXCL11* (forward), 5’-GACGCTGTCTTTGCATAGGC-3’; and (reverse), 5’-GGATTTAGGCATCGTTGTCCTTT-3’; *CCL17* (forward), 5’-CCCCAGACTCCTGACTGTCT-3’; and (reverse), 5’-TCTCCCTCACTGTGGCTCTT-3’; *CCL20* (forward), 5’-ACTGGGTACTCAACACTGAGC-3’; and (reverse), 5’-CAAAGCAGCCAGGAGCAAAC-3’; *CCL22* (forward), 5’-GCCTACTCTGATGACCGTGG-3’; and (reverse), 5’-AGAGAGTTGGCACAGGCTTC-3’; *IL-33* (forward), 5’-GTGACGGTGTTGATGGTAAGAT-3’; and (reverse), 5’-AGCTCCACAGAGTGTTCCTTG-3’; *IL1RL1* (forward), 5’-GAAAACCTAGTTACACCGTGGAT-3’; and (reverse), 5’-GCAAACACACGATTTCTTTCCTG-3’; *GAPDH* (forward), 5’-ACAACTTTGGTATCGTGGAAGG-3’; and (reverse), 5’-GCCATCACGCCACAGTTTC-3’.

### Transwell Migration Assay

The migration of Tregs and Tconvs was assessed using a 5-µm pore size transwell insert system (Corning, New York, USA). FACS-sorted Tregs and Tconvs were pre-treated with C 021 dihydrochloride (a CCR4 antagonist) (100 nmol/L) (Tocris, Minneapolis, MN, USA) for 30 minutes and plated in the upper chambers of transwell inserts. Medium with or without supplementation with recombinant CCL17 (10 ng/mL) (PeproTech) was placed in the lower chambers. After incubation for 12 hours at 37°C in 5% CO_2_, cells were harvested from the bottom chambers and counted with a hemocytometer.

### Enzyme-Linked Immunosorbent Assay (ELISA) and Luminex Assay

Plasma samples were aliquoted and stored at −80°C until analysis. The enhanced liver fibrosis (ELF) score is composed of hyaluronic acid (HA), tissue inhibitor of matrix metalloproteinase-1 (TIMP-1) and amino-terminal propeptide of procollagen type III (PIIINP) (18). Plasma concentrations were measured using quantitative ELISAs for HA, TIMP-1 (R&D Systems) and PIIINP (USCN Life, Wuhan, China) according to the manufacturers’ instructions.

The concentration of IL-33 was detected in duplicate using a Human Magnetic Luminex Assay (R&D Systems, Minneapolis, MN, USA) according to the manufacturer’s instructions. The measurement was performed in a Luminex 200 system (Luminex, Austin, TX, USA).

### Statistical Analysis

Categorical variables are presented as numbers and percentages, and continuous variables are expressed as mean ± standard deviation (SD) values unless otherwise indicated. Statistical analysis was performed with GraphPad Prism software 8.0 (GraphPad Software, La Jolla, CA). Unpaired and paired t tests were used to perform comparisons between two groups. One-way or two-way ANOVA with the Sidak *post hoc* test was used to compare means among more than two groups. Correlations were assessed with Spearman’s correlation coefficient. A value of *P <*0.05 was considered statistically significant.

## Results

### Circulating and Orbit-Infiltrating Tregs Were Expanded in IOI

**Figure 1:**
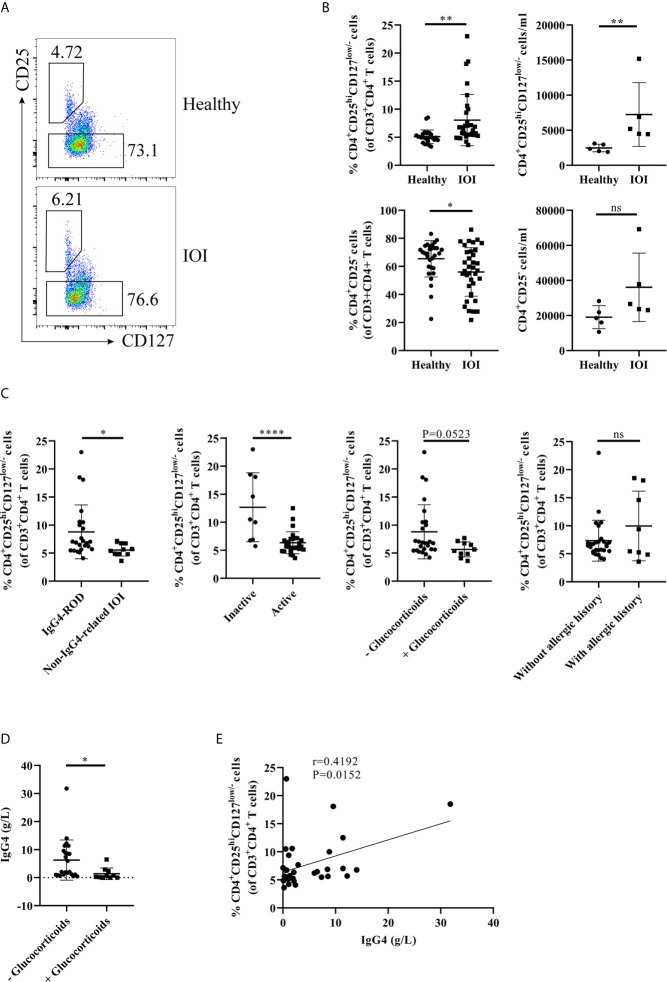
The frequency of circulating regulatory T cells (Tregs) was increased in idiopathic orbital inflammation (IOI). The frequencies of Tregs in patients with IOI and healthy control subjects were evaluated by flow cytometry. **(A, B)** The frequencies and absolute number of circulating Tregs and conventional T cells (Tconvs) in healthy controls (n=30) and patients with IOI (n=36). **(C)** Frequencies of circulating Tregs compared between patients stratified by IgG4-related disease (IgG4-RD), disease activity, allergic history and glucocorticoid therapy history status. **(D)** Compare the serum IgG4 between IOI patients with and without a history of glucocorticoids. **(E)** Association between circulating Tregs and serum IgG4 levels in patients with IOI. *P < 0.05; **P < 0.01; ****P < 0.0001; ns, not significant.

**Figure 2:**
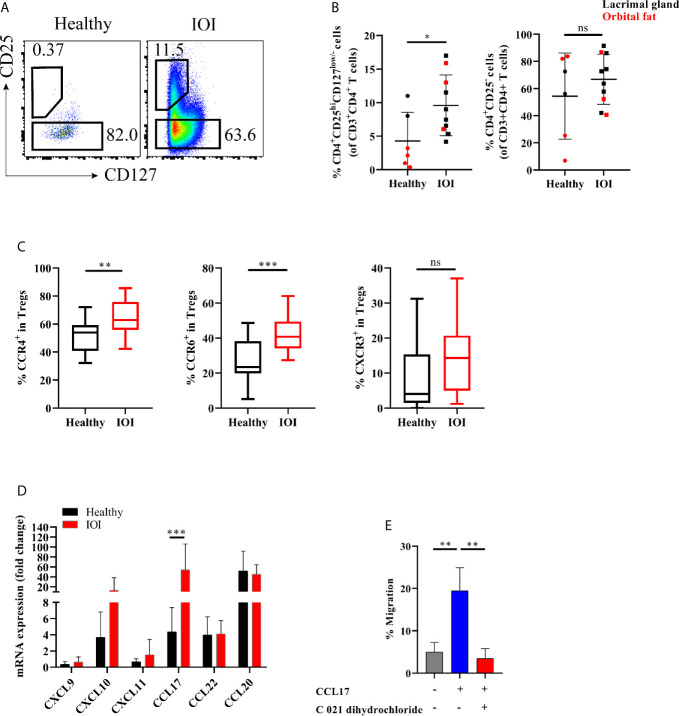
Expanded orbit-derived regulatory T cells (Tregs) in patients with idiopathic orbital inflammation (IOI). The frequency of orbit-infiltrating Tregs was analyzed by flow cytometry. **(A, B)** Frequency of orbit-infiltrating Tregs in patients with IOI (n=10) and healthy controls (n=6). **(C)** The proportions of CCR6^+^ Tregs, CCR4^+^ Tregs and CXCR3^+^ Tregs in blood were calculated. **(D)** CXCL9, CXCL10, CXCL11, CCL17, CCL20 and CCL22 mRNA expression in orbital fat from healthy controls and patients with IOI. **(E)** Migration of Tregs cultured in the presence of CCL17 or pretreated with CCR4 antagonist (C 021 dihydrochloride). *P < 0.05; **P < 0.01; ***P < 0.001; ns, not significant.

Initially, we carried out flow cytometry to assess the frequencies of circulating and orbit-infiltrating Tregs in healthy individuals and patients with IOI. Tregs and Tconvs were classified as CD4^+^CD25^hi^CD127^low/-^ cells and CD4^+^CD25^-^ cells, respectively (**Figures 1A**, **2A**). As shown in **Figure 1B**, compared to that in healthy controls, the proportion and absolute number of circulating Tregs were significantly elevated in patients with IOI (both *P* < 0.01). Parallel analysis of Tconvs revealed that the frequency of peripheral Tconvs was decreased in patients with IOI (*P* < 0.01), while there were no significant differences between IOI patients and healthy subjects in the absolute number of circulating Tregs (*P* > 0.05). Among IOI patients, no difference was noted between those with a history of allergy (*P*>0.05), but the frequency of circulating Tregs was reduced in the active subgroup compared with that in inactive subgroup (*P* < 0.0001). Notably, the frequency of Tregs was significantly higher in patients with IgG4-ROD than in those with non-IgG4-related IOI (*P* < 0.05). Moreover, IOI patients with a history of glucocorticoids showed a tendency to be decreased in the percentage of Tregs (*P*=0.0523) (**Figure 1C**). The level of serum IgG4 was significantly lower in IOI patients with a history of glucocorticoids than those without a history of glucocorticoids (*P*<0.05) (**Figure 1D**). Additionally, a significantly positive correlation was found between the Treg frequency and serum IgG4 level (r=0.4192, *P*=0.0152) (**Figure 1E**).

Next, we investigated whether the frequency of Tregs in the inflamed orbit was altered. The frequency of orbit-derived Tregs was obviously elevated in patients with IOI compared with healthy subjects (*P <*0.05), while the frequency of Tconvs did not differ significantly between IOI patients and healthy controls (*P* > 0.05) (**Figure 2B**). We then evaluated the expression of chemokine receptors in peripheral Tregs. CCR4 and CCR6, expressed in circulating Tregs, were upregulated in patients with IOI (*P* < 0.01 and *P* < 0.001, respectively) (**Figure 2C**). In contrast, the CCR4^+^ and CCR6^+^ compartments among orbit-derived Tregs from patients with IOI were decreased (*P* < 0.01 and *P* < 0.05, respectively) (**Figures S1A, B**). However, the frequency of CXCR3-expressing Tregs was unchanged in our cohort of IOI patients (*P* > 0.05) (**Figures S1C**).

We next examined the expression of CCL17 and CCL22 (CCR4 ligands); CXCL9, CXCL10 and CXCL11 (CXCR3 ligands); and CCL20 (CCR6 ligand) in explants from IOI patients and controls. Consistent with the expression patterns of chemokine receptors, affected orbital tissues from patients with IOI exhibited increased expression of CCL17 (*P* < 0.001) (**Figure 2D**). To further ascertain whether CCL17 facilitates the migration of circulating Tregs to inflamed orbital tissues, we performed a transwell assay of circulating Tregs treated with or without CCR4 antagonist (C 021 dihydrochloride) in the presence of CCL17. Notably, CCL17 enhanced the chemotaxis of circulating Tregs from patients with IOI (*P* < 0.01), and this effect was attenuated by treatment with the CCR4 antagonist (*P* < 0.01) (**Figure 2E**).

### IOI Tregs Exhibited Diminished Suppressive Capacity

**Figure 3:**
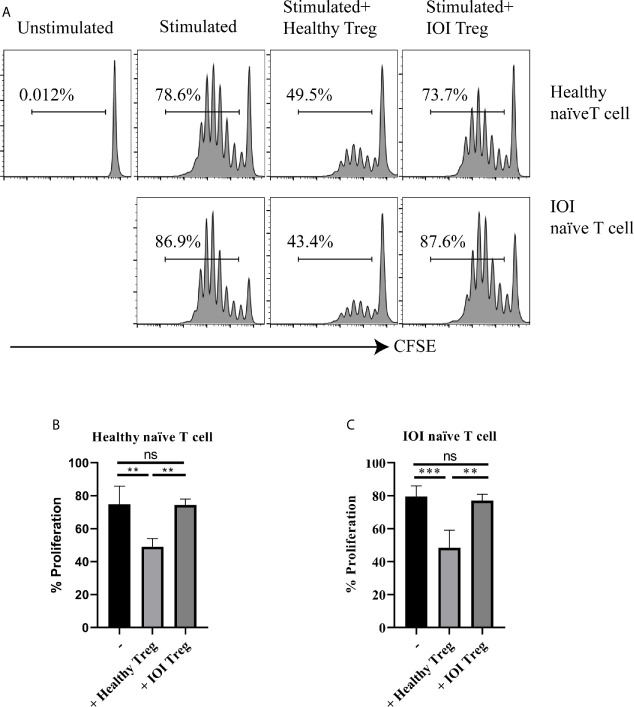
The suppressive function of Tregs invitro. Sorted-Tregs were cocultured with CFSE labeled naïve T cells. The proliferation of naïve T cells were examined after 5 days. **(A)** is one representative image. **(B, C)** is statistical images. **P < 0.01; ***P < 0.001; ns, not significant.

We further evaluated the suppressive capacity of Tregs in patients with IOI. As shown in **Figures 3A–C**, whether cocultured with healthy naïve T cells or IOI naïve T cells in the presence of anti-CD3/anti-CD28 and IL-2, Tregs from healthy subjects significantly decreased the proliferation of naïve T cells (*P* < 0.01 and *P* < 0.001, respectively), whereas Tregs from IOI patients could not inhibit the proliferation of naïve T cells (both *P* > 0.05), suggesting that Tregs from IOI patients are dysfunctional, rather than naïve T cells are resistant to Treg suppression.

In addition, we measured the cytokines to assess the suppressive function of Tregs indirectly. First, we evaluated the baseline levels of inflammatory cytokines produced by Tconvs. As shown in **Figures S2A–D**, the levels of TNF-α, IFN-γ, and IL-17A secreted from Tconvs were comparable between IOI and healthy controls (all *P* > 0.05), whereas the IL-4-producing Tconvs tend to be increased in IOI patients. We further grouped the patients according to their allergic history, and found that IL-4-producing Tconvs were higher in IOI patients with allergic history than those without allergic history (*P* < 0.05) (**Figure S2E**). Coculture of Tconvs with autologous Tregs from healthy individuals led to a clear reduction in proinflammatory TNF-α^+^CD4^+^ (*P* < 0.05) and IFN-γ^+^CD4^+^ T cells (*P* < 0.05). However, Tregs from patients with IOI did not exhibit altered production of TNF-α^+^CD4^+^ and IFN-γ^+^CD4^+^ T cells (both *P* > 0.05) (**Figures S3A, B**). In both healthy controls and patients with IOI, the frequencies of IL-17A^+^CD4^+^ and IL-4^+^CD4^+^ T cells did not differ between Tconvs cocultured without and with autologous Tregs (all *P* > 0.05) (**Figures S3C, D**).

### Tregs Transdifferentiated Into Proinflammatory and Profibrotic Phenotypes in IOI

**Figure 4:**
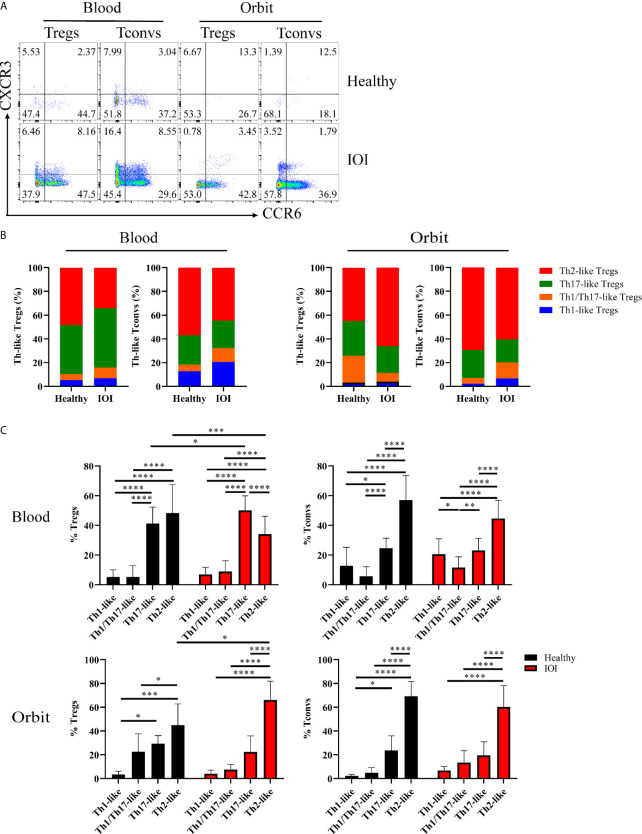
Distribution of Th-like regulatory T cells (Tregs) in healthy subjects and patients with idiopathic orbital inflammation (IOI). **(A)** Representative plots of chemokine receptor CCR6 and CXCR3 expression in CCR4+ Tregs and conventional T cells (Tconvs). **(B, C)** Percentages of Th-like Tregs in peripheral blood (healthy, n = 15; IOI, n = 21) and orbit (healthy, n = 4; IOI, n = 5). *P < 0.05; **P < 0.01; ***P < 0.001; ****P < 0.0001.

In the following experiment, we determined the distributions of Th-like Treg and Tconv subsets in peripheral blood and orbital tissues from healthy individuals and patients with IOI. Th-like Treg and Tconv subsets can be identified based on distinctive expression patterns of the chemokine receptors CXCR3, CCR4, and CCR6 (**Figure S4**). To clarify whether the Treg phenotype was altered, we compared the expression of CXCR3 and CCR6 among CCR4^+^ Tregs and CCR4^+^ Tconvs in blood and orbital tissues between healthy subjects and IOI patients (**Figure 4A**). The Th2-like subset accounted for the highest proportions (both approximately 40-70%) of CCR4^+^ Tregs and CCR4^+^ Tconvs in peripheral blood from healthy subjects and normal orbital fat tissues (**Figure 4B**). The frequency of circulating Th17-like Tregs was elevated in IOI patients (*P* < 0.05), whereas that of circulating Th2-like Tregs was significantly decreased (*P* < 0.001). Intriguingly, the frequency of Th2-like Tregs was elevated in orbital tissues compared with normal tissues (*P* < 0.05) (**Figure 4C**). We retrospectively compared Treg phenotypes in patients with and without a history of glucocorticoids therapy. Surprisingly, our data showed that glucocorticoids did not affect the phenotype of Tregs in IOI patients (all *P* > 0.05) (**Figure S5**). Foxp3 acts as a lineage specification factor essential for the differentiation of Tregs. The expression of Foxp3 was positively correlated with Th1/Th17-like Treg (r=0.6956, *P* < 0.01), while negatively associated with Th2-like Treg (r=-0.5003, *P* < 0.05) (**Figure S6**).

**Figure 5:**
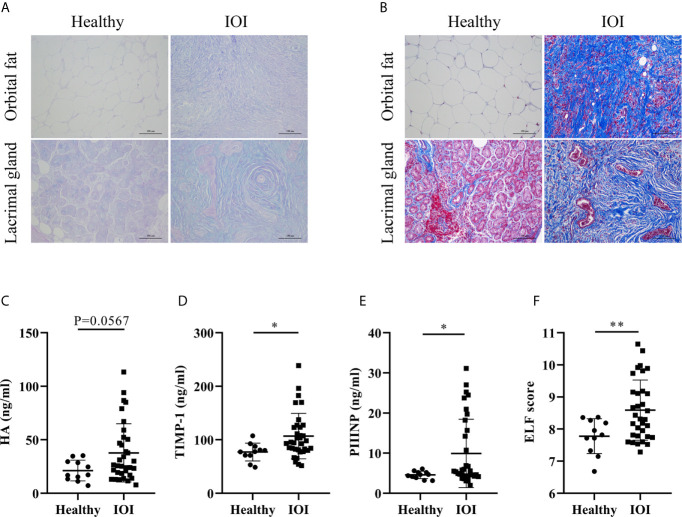
Degree of fibrosis in idiopathic orbital inflammation (IOI). **(A)** Immunohistochemical staining of hyaluronic acid (HA) (Alcian blue/periodic acid-Schiff (AB-PAS) staining, magnification ×200) and **(B)** collagen (Masson staining, magnification ×200). Concentrations of plasma HA **(C)**, matrix metalloproteinase-1 (TIMP-1) **(D)** and amino-terminal propeptide of procollagen type III (PIIINP) **(E)**. **(F)** Enhanced liver fibrosis (ELF) scores of patients with IOI (n = 33) and healthy controls (n = 11). *P < 0.05; **P < 0.01.

Considering that excessive Th2-like Tregs might enhance skin fibrosis in fibrotic tissues (19), we evaluated the severity of tissue fibrosis in IOI patients based on histologic staining of extracellular matrix (ECM) deposition and the ELF score, a clinically validated surrogate marker of tissue fibrosis (18, 20). Our results indicated increases in HA (AB-PAS staining, blue) and collagen (Masson staining, blue) in orbital fat and lacrimal glands of IOI patients (**Figures 5A, B**). Moreover, the concentrations of HA (*P*=0.0567), TIMP-1 (*P* < 0.05) and PIIINP (*P* < 0.05) were elevated in patients with IOI (**Figures 5C–E**), and the corresponding ELF scores were higher in patients with IOI than in healthy subjects (*P* < 0.01) (**Figure 5F**).

We further analysis the association between circulating Th-like Treg subsets and plasma fibrosis marker levels. There was no significant correlation between the frequency of Tregs and plasma fibrosis marker levels (all *P* > 0.05) (**Figure S7A**), but circulating Th2-like Tregs were positively correlated with the level of plasma HA and ELF score (both *P* < 0.05) (**Figure S7B**), suggesting that the fibrotic orbit in patients with IOI may be driven by Tregs that have transdifferentiated into profibrotic Th2-like phenotype.

### IL-33 Suppressed the Proinflammatory and Profibrotic Function of Tregs in IOI

**Figure 6:**
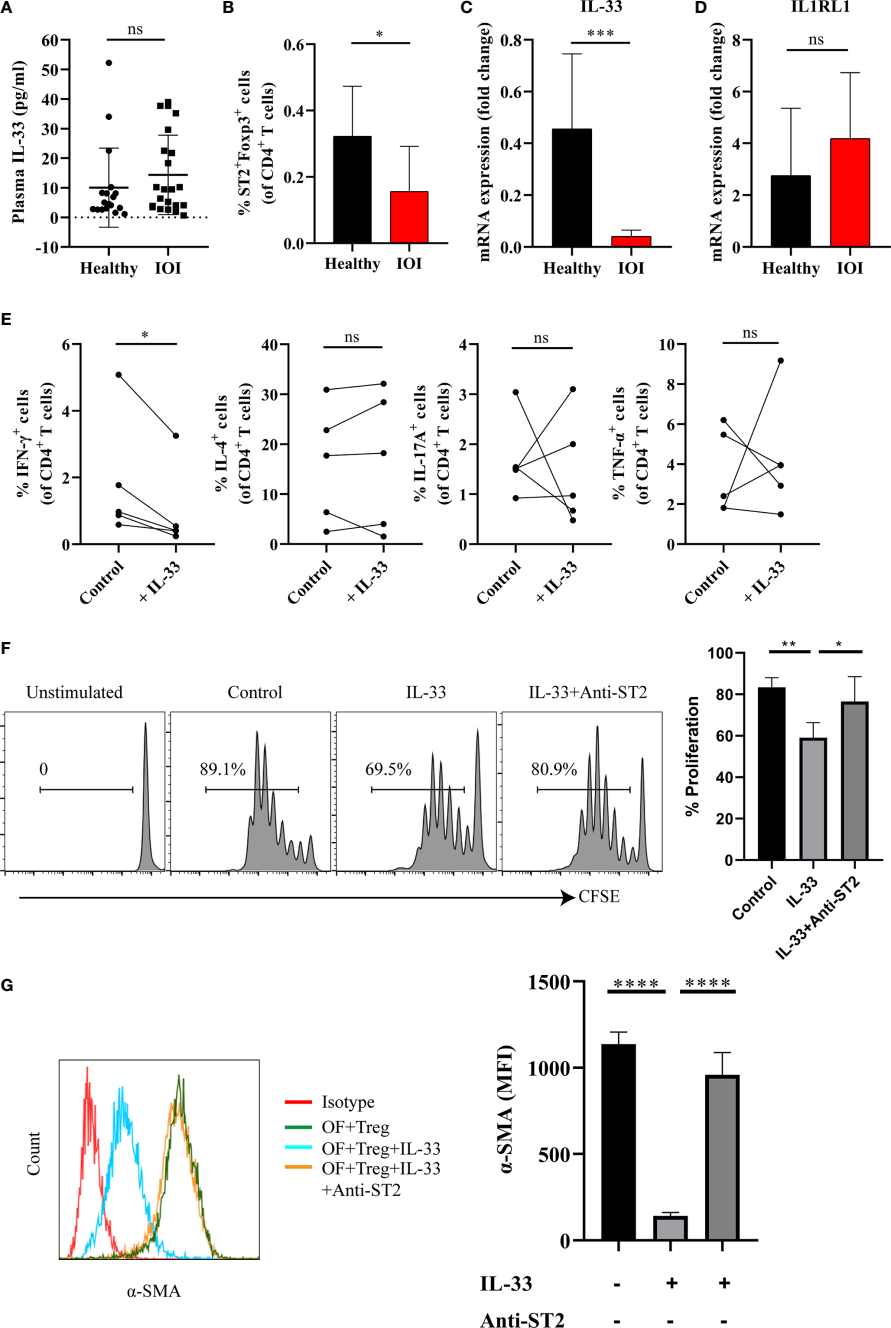
IL-33 restored the suppressive function of Tregs, inhibited the production of interferon (IFN)-γ by regulatory T cells (Tregs) and the activation of orbital fibroblasts (OFs) in idiopathic orbital inflammation (IOI). **(A)** The plasma level of interleukin (IL)-33 in healthy controls (n = 18) and patients with IOI (n = 22) was assessed by Luminex technology. **(B)** The proportion of circulating ST2^+^Foxp3^+^ Tregs in healthy controls (n = 14) and patients with IOI (n = 8). **(C)** IL-33 and **(D)** IL1RL1 mRNA expression in orbital fat from healthy subjects (n = 4) and patients with IOI (n = 15). **(E)** Intracellular cytokines secreted by FACS-sorted Tregs from IOI patients (n = 5) were detected after stimulation with IL-33 in the presence of anti-CD3 and anti-CD28 and IL-2. **(F)** Proliferation of CFSE labeled naïve T cells cocultured with sorted-Tregs under different treatment conditions. **(G)** Expression of a-SMA in OFs cocultured with Tregs under different treatment conditions. *P < 0.05; **P < 0.01; ***P < 0.001; ****P < 0.0001; ns, not significant.

To identify the factor that drives the proinflammatory function of Tregs, we shifted our focus to IL-33 and its receptor IL1RL1 (also called ST2). As shown in **Figure 6A**, there was no significant difference in the level of plasma IL-33 between IOI patients and healthy controls (*P* > 0.05). However, a significantly decreased proportion of circulating Foxp3^+^ Tregs coexpressed ST2 was found in patients with IOI (*P* < 0.05) (**Figure 6B**). Additionally, in patients with IOI, the IL-33 mRNA level in orbital tissues was decreased (*P* < 0.001) (**Figure 6C**), although the level of IL1RL1 mRNA was unchanged (*P* > 0.05) (**Figure 6D**). However, there was no significant correlation between circulating Tregs and the level of plasma IL-33 (**Figure S8**). We then assessed whether IL-33 impact the function of suppressive Tregs in IOI. Our results showed that IFN-γ secreted from Tregs was significantly decreased (*P* < 0.05) in the presence of IL-33 (**Figure 6E**), whereas IL-33 did not affect the production of IFN-γ, IL-4, IL-17A and TNF-α from Tconvs (all *P* > 0.05) (**Figure S9**). Likewise, IL-33 significantly inhibited the proliferation of naïve T cells (*P* < 0.01), while blocking ST2 counteracted this inhibition (*P* < 0.05) (**Figure 6F**).

Furthermore, to clarify whether IL-33 exerts antifibrotic or profibrotic effects in IOI, we cocultured orbital fibroblasts (OFs) (act as fibrotic effector cells) with Tregs were stimulated with IL-33, and assessed OFs activation status by detecting α-smooth muscle actin (α-SMA). As shown in **Figure 6G**, the expression of α-SMA was significantly attenuated when cocultured OFs and Tregs were treated with IL-33 (*P* < 0.0001), whereas blockade of ST2 restored the activation of OFs (*P* < 0.0001). Additionally, we stimulated OFs with different concentrations (0, 1, 10, 20, 50, 100 ng/ml) of IL-33 for 24, 48, and 72 hours. We found that the mean fluorescence intensity of ST2 and α-SMA in OFs did not changed when stimulated with the different concentrations of IL-33 (all *P* > 0.05) (**Figure S10**). Collectively, our results suggested that the regulatory role of IL-33 is specific to Tregs, but not to Tconvs or OFs.

## Discussion

Treg-mediated suppression plays an indispensable role in immune-mediated inflammation, autoimmune disorders, allergies, infections, cancers, and metabolic inflammation (6). Decreased, increased and unchanged frequencies of peripheral Tregs have been reported in patients with various systemic autoimmune diseases (21). In this study, expansion of Tregs in peripheral blood and affected orbital tissues was observed in patients with IOI, consistent with previous reports in patients with IgG4-RD (8, 22). In line with previous study (23), our results confirmed that the frequency of Tregs were increased in patients with IOI and positively correlated with the serum levels of IgG4. IL-10, which is primarily produced by Tregs, enhances IgG4 production by promoting IL-4-induced IgG4 immunoglobulin class-switching (24). Therefore, the increased Tregs from IOI patients may induce IgG4 class-switching by producing more IL-10, which potentiates the production of serum IgG4. Here, the possibly reflexive elevation of Tregs in IOI may be caused by the response of Tregs to persistent inflammation. However, the mechanism of Treg recruitment into the inflamed orbit remains unknown. Chemokine-induced trafficking and recruitment of leukocytes is involved in the inflammatory response (25), and chemokine receptors on Tregs are involved in the recruitment of these cells to sites of persistent inflammation (26, 27). Recent study indicated that CCR4 and CXCR3 may participate in psoriasis recurrence or redistribution to distant sites (28). Therefore, we proposed that the accumulation of orbit-infiltrating Tregs in IOI may be due to the elevated expression of CCR4/CCR6 in circulating Tregs and increased levels of the corresponding ligand CCL17 in tissues, which might induce the homing of circulating Tregs into the orbit in patients with IOI.

Classically, Tregs constitute a lineage of T lymphocytes with immunosuppressive functions, and maintenance of Foxp3 expression is central to Treg lineage stability (29). However, accumulating evidence indicates that Tregs are a highly dynamic set of immune cells, with an orchestrated balance under different environmental contexts that tailors their functions and homeostatic properties to a wide range of immune niches (30). Plasticity is a property inherent to most immune cells, including Tregs. Inflammatory conditions endow Tregs with a functional shift to a proinflammatory phenotype (12, 31, 32). Reprogramming of Tregs toward Th17 plasticity was found in patients with RA and patients with systemic lupus erythematosus (13, 15). In addition, IFN-γ^+^ Tregs and Th2-like Tregs were found to be enriched in patients with type 1 diabetes and food allergy, respectively (9, 32). Therefore, we hypothesized that Treg plasticity might contribute to the pathogenesis of IOI. Here, we provided a comprehensive analysis of the coexpression of cytokine receptors by Th-like Tregs. Circulating Tregs were polarized to a Th17-like phenotype in patients with IOI, whereas orbit-derived Tregs were reprogrammed toward a Th2-like lineage. These discrepancies may be related to the different roles of Tregs in peripheral blood and their local organization.

Pathogenic Th1, Th2 and Th17 responses are suppressed by Tregs (33). However, insights into the function of Tregs in patients with IOI are limited. Prior work has established a proinflammatory role for peripheral Tregs in autoimmune diseases (9, 13). Consistent with this role, our results showed that aberrant reprogramming of peripheral Tregs in patients with IOI resulted in diminished suppressive capacity, which was accompanied by a feedback-mediated increase in the Treg frequency. Because of the insufficient number of Tregs from normal orbital tissues, we could not examine whether Tregs from the inflamed orbit are functionally impaired. We assumed that orbit-derived Tregs may play a fibrotic role, based on their Th2-like phenotype.

IL-33, a member of the IL-1 superfamily of cytokines, has been shown to decrease IL-17 and IFN-γ production in an experimental autoimmune encephalomyelitis (EAE) model (34, 35). IL1RL1 activates the MyD88/NF-κB signaling pathway to promote the suppressive effect of Tregs in inflammatory diseases (36), but the role of IL-33 in autoimmune diseases and tumors is controversial (36–38). IL-33 signaling enhances the expression of ST2 in Tregs (36), and ST2 expression is associated with an amelioration of the inhibitory function of Tregs (39); hence, IL-33 signaling increases the protective effect of Tregs in shaping an immunosuppressive environment (40, 41). However, the question of this signaling can also affect IOI is unanswered. The present discovery provided new evidence that IL-33/ST2 signaling promotes the immunoregulatory microenvironment in IOI. First, IL-33/ST2 signaling was downregulated in IOI. Second, exogenous IL-33 stimulation reduced the production of IFN-γ by Tregs and restored the immunosuppressive function of Tregs, indicating that IL-33 may exert anti-inflammatory effects on the process of IOI.

Human Tregs have been extensively studied in blood, but interest in the role of tissue-resident Tregs is increasing (42). Tregs contribute to the maintenance of tissue homeostasis by promoting wound healing and repair processes in numerous tissues (43). Although we could not sort sufficient numbers of Tregs from normal orbital connective tissues for functional analysis, we isolated tissue-resident Tregs for phenotype analysis. Tregs accumulate and become skewed toward a Th2 phenotype in IOI masses, as has been reported in systemic sclerosis (19), melanoma and colorectal cancer (44), indicating that Th2-skewed Tregs are preferentially found in fibroproliferative orbital tissues. Thus, our collective results emphasize the core identity of resident Tregs in fibrosis. In addition, Exogenous IL-33 attenuated the expression of α-SMA when cocultured OFs with Tregs indicating that IL-33 may play an anti-fibrotic role in patients with IOI.

The mainstay treatment for IOI is glucocorticoid therapy, which nonspecifically suppresses inflammation (45). Recent reports have suggested that rituximab (RTX) therapy is effective for refractory cases, but other patients remain unresponsive to therapy or relapse (17, 46). Tregs play a role in suppressing effective immunity and tissue homeostasis (6, 47) and are thus an attractive therapeutic candidate. Treg therapy has been proven to be effective in patients with graft-versus-host disease, type 1 diabetes and organ transplantation (48). As mentioned above, dysfunctional Tregs contribute to inflammatory disease, and our data suggest that Tregs in IOI are proinflammatory and profibrotic. Tregs have potential as multifaceted adaptable therapeutics and could be used as living drugs, including IL-2 therapy, adoptive cell therapy (ACT), chimeric antigen receptor (CAR) Treg therapy, or co-medication (49). In our study, Tregs in IOI were proinflammatory and profibrotic. IL-33 restored the suppressive function of Tregs, reduced the OF activation and IFN-γ production *via* Tregs, rather than Tconvs or OFs. Therefore, targeted Treg therapy coupled with IL-33 therapy might be a potential option for IOI patients.

The study does have limitations due to the difficulty in recruiting patients with IOI and the specimen volume was small. First, since we could not collect enough extraocular muscles (EOMs) to analyze the distribution and function of Tregs. Additionally, specimens from affected orbit contained insufficient Tregs for further functional assay.

In conclusion, our data characterized the distribution and function of plastic Tregs in patients with IOI. We demonstrated herein that both circulating and orbit-infiltrating Tregs were expanded in IOI. Importantly, proinflammatory and profibrotic Tregs may lead to the development of IOI. In addition, IL-33 can suppress the proinflammatory and profibrotic effect of Tregs. These findings suggest reversing the plastic Tregs *via* IL-33 as an appealing therapeutic target for the resolution of inflammation and fibrosis in IOI, but further studies are warranted for a more detailed exploration.

## Data Availability Statement

The raw data supporting the conclusions of this article will be made available by the authors, without undue reservation.

## Ethics Statement

The studies involving human participants were reviewed and approved by the Institutional Review Board of Zhongshan Ophthalmic Center, Sun Yat-sen University. The patients/participants provided their written informed consent to participate in this study.

## Author Contributions

JC, HYe, and WX designed experiments and wrote the manuscript. YM, SA, RC, and XL recruitment donors. JC, LS, XW, and SB conducted data acquisition. SY, XJ, and TZ performed experiments. HYa directed and supervised the study. All authors contributed to the article and approved the submitted version.

## Funding

The National Natural Science Foundation of China (no. 81700875, no. 81870689, no. 81800866 and no. 81670887) and The Fundamental Research Funds of the State Key Laboratory of Ophthalmology. We thank the Core Facilities at State Key Laboratory of Ophthalmology, Zhongshan Ophthalmic Center for technical support.

## Conflict of Interest

The authors declare that the research was conducted in the absence of any commercial or financial relationships that could be construed as a potential conflict of interest.
