# Supplementary material for: Increased Dysfunctional and Plastic Regulatory T Cells in Idiopathic Orbital Inflammation
Source: Front Immunol. 2021 May 3;12:634847. doi: 10.3389/fimmu.2021.634847 (PMC8126653; doi:10.3389/fimmu.2021.634847)
Supplement: Supplementary file 1 [file DataSheet_1.docx]

Table S1. Characteristics of Participants

|  | Healthy  (n=58) | IOI  (n=67) | *P*-value |
| --- | --- | --- | --- |
| Age (y), mean ± SD | 39.91 ± 16.20 | 42.24 ± 14.58 | 0.4001 |
| Sex (n, %) |  |  | 0.8577† |
| Female | 34 (47.22) | 38 (52.78) |  |
| Male | 24 (45.28) | 29 (54.72) |  |
| IgG4/HPF, median (IQR) | - | 30.00 (10.00-60.00) | - |
| IgG/HPF, mean ± SD | - | 58.60 ± 28.94 | - |
| IgG4/IgG, median (IQR) | - | 60.00 (25.00-80.00) | - |
| IgG4(g/L), median (IQR) | - | 1.89 (0.66-8.51) | - |
| C3(g/L), median (IQR) | - | 1.19 (1.02-1.42) | - |
| C4(g/L), median (IQR) | - | 0.26 (0.20-0.33) | - |

IOI=idiopathic orbital inflammation, SD=standard deviation, IQR=interquartile range.

†Chi-square test.


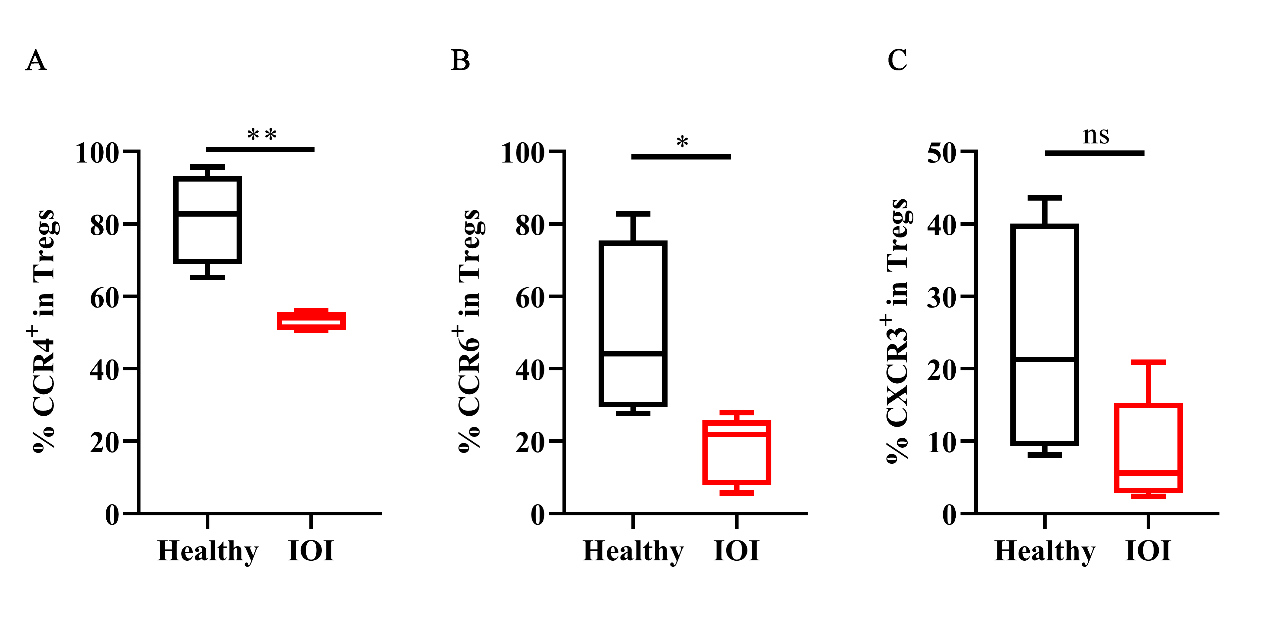


**Fig S1.** Expression of CCR6, CCR4 and CXCR3 in orbit-infiltrating regulatory T cells (Tregs). Orbit-derived Tregs from healthy subjects (n=4) and patients with idiopathic orbital inflammation (IOI) (n=5) were stained for CCR4 (A), CCR6 (B) and CXCR3 (C). **P* < 0.05; ***P* < 0.01; ns, not significant.


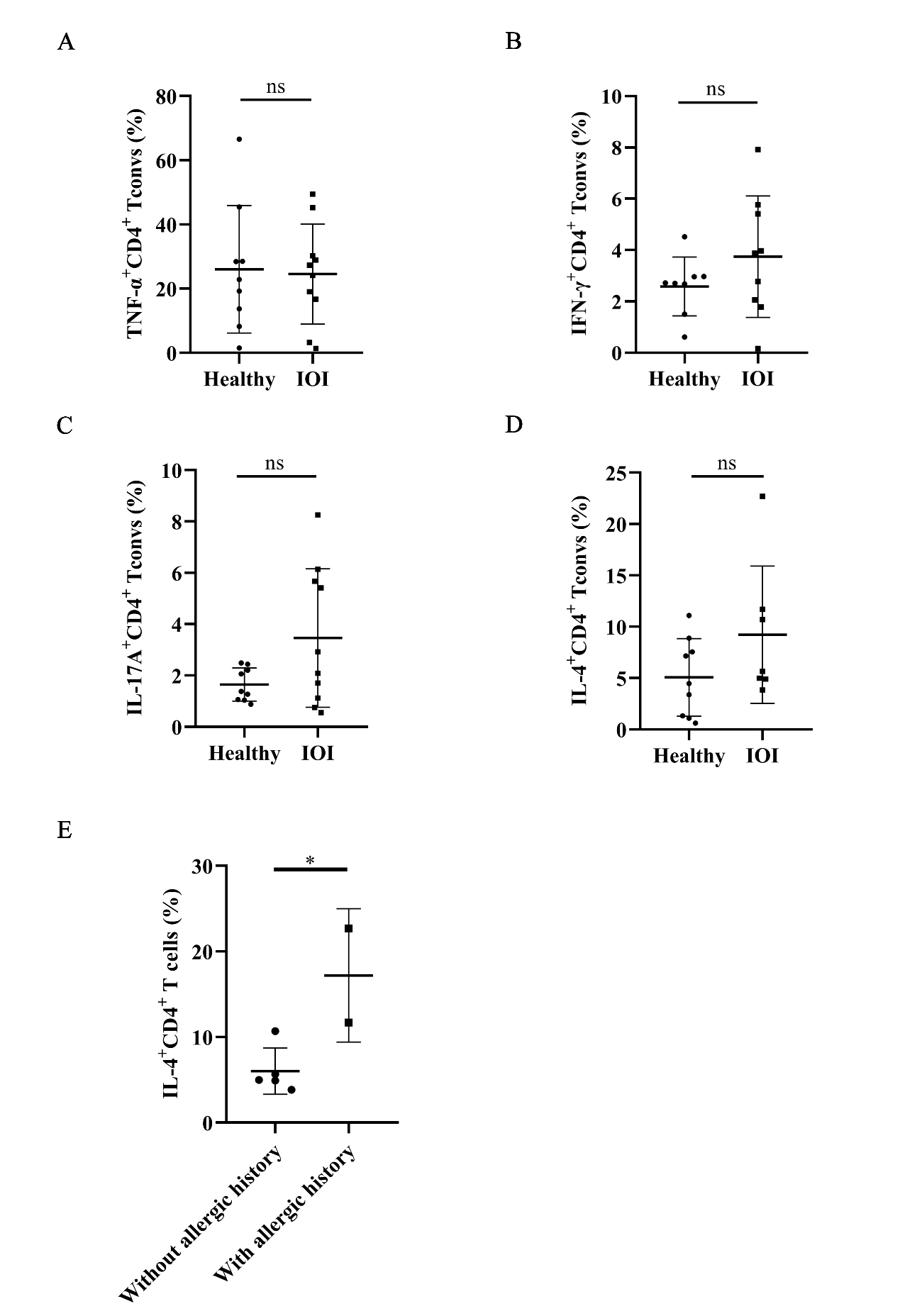


**Fig S2.** Cytokine production profiles of peripheral conventional T cells (Tconvs) in healthy controls (n=8-9) and patients with idiopathic orbital inflammation (IOI) (n=7-10). Peripheral Tconvs were cultured in the presence of anti-CD3 and anti-CD28 and IL-2. The expression of tumor necrosis factor (TNF)-α (A), interferon (IFN)-γ (B), interleukin (IL)-17A (C) and IL-4 (D) in CD4+ T cells was assessed by intracellular cytokine staining and flow cytometry. (E) Compare IL-4-producing Tconvs in IOI patients with allergic history and those without allergic history. ns, not significant.


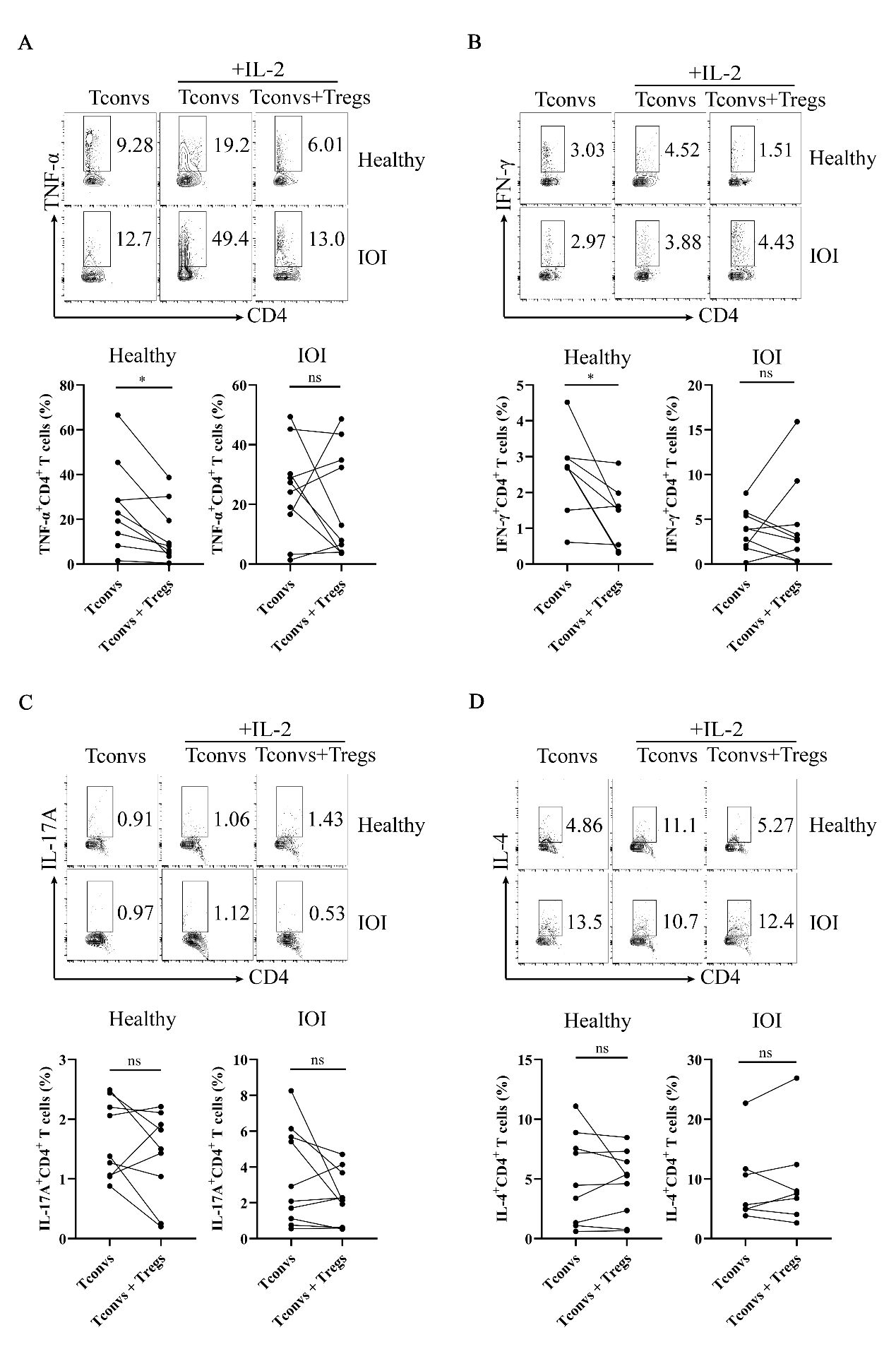


Fig S3. The suppressive function of peripheral regulatory T cells (Tregs) was impaired in patients with idiopathic orbital inflammation (IOI). Peripheral conventional T cells (Tconvs) cultured with or without autologous Tregs in vitro in the presence of anti-CD3 and anti-CD28 and interleukin (IL)-2. The expression of tumor necrosis factor (TNF)-α (A), interferon (IFN)-γ (B), IL-17A (C) and IL-4 (D) in CD4^+^ T cells was assessed by intracellular cytokine staining and flow cytometry in samples from healthy subjects (n=8-9) and patients with IOI (n=7-10). **P* < 0.05; ns, not significant.


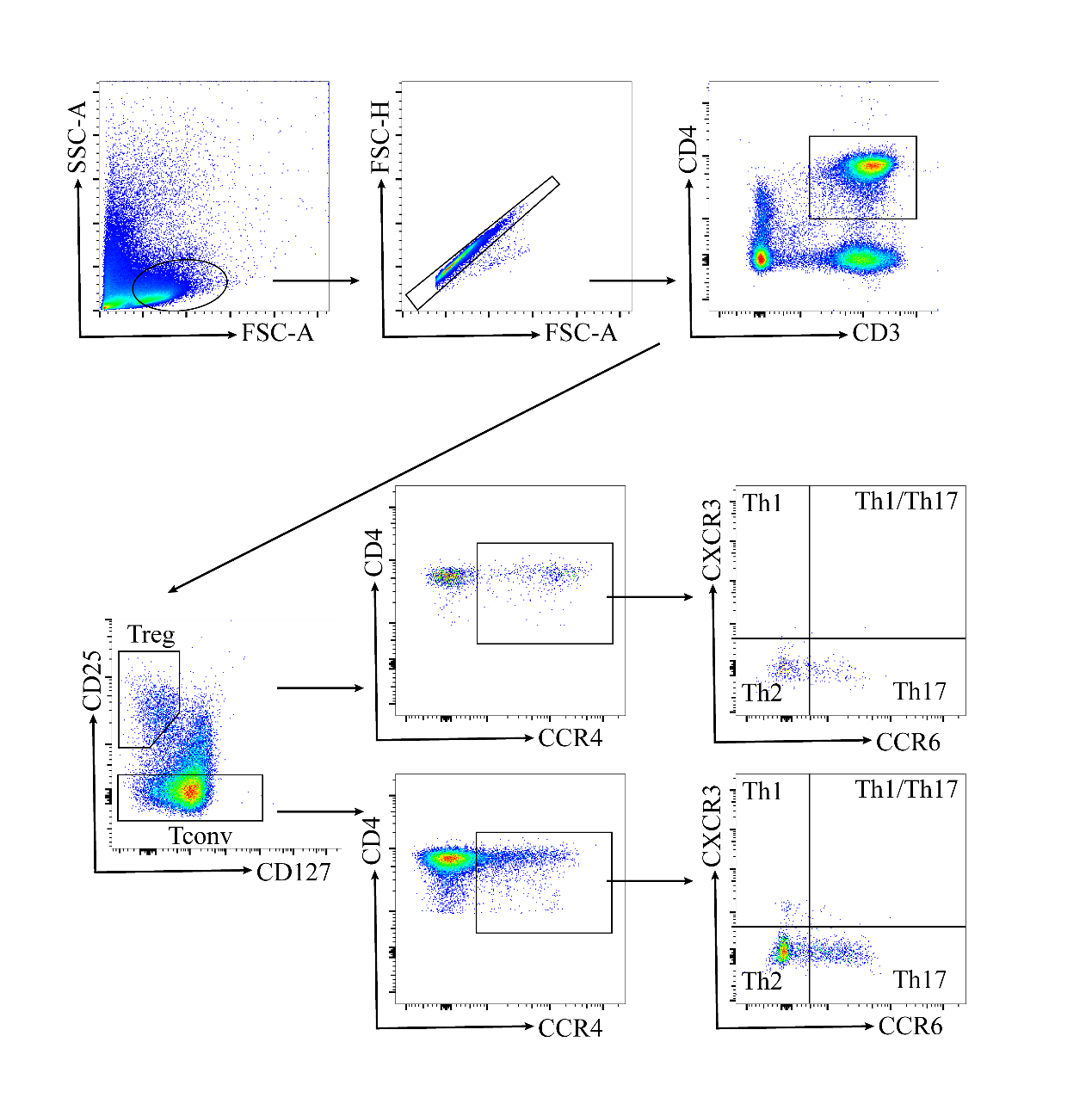


**Fig S4.** Gating strategy for the identification of Th-like regulatory T cells (Tregs) and Th-like conventional T cells (Tconvs) in peripheral blood and orbit explants.


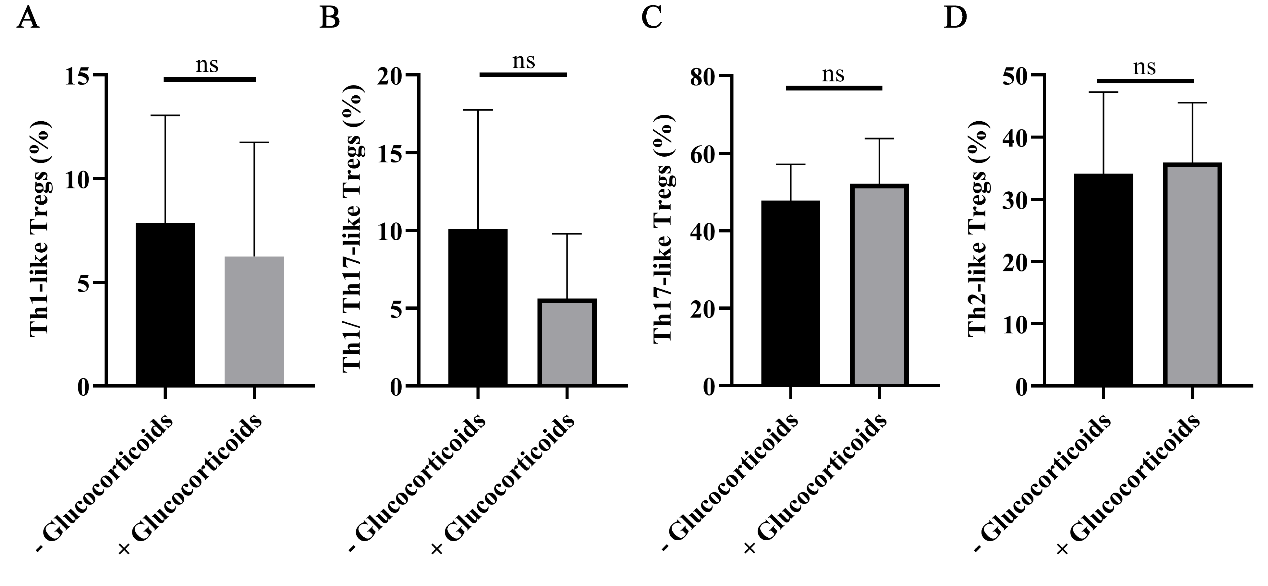


**Fig S5.** The effect of glucocorticoids on the phenotype of Tregs. (A), (B), (C), (D) represent the Th1-like, Th1/Th17-like, Th17-like, and Th2-like phenotype, respectively. ns, not significant.


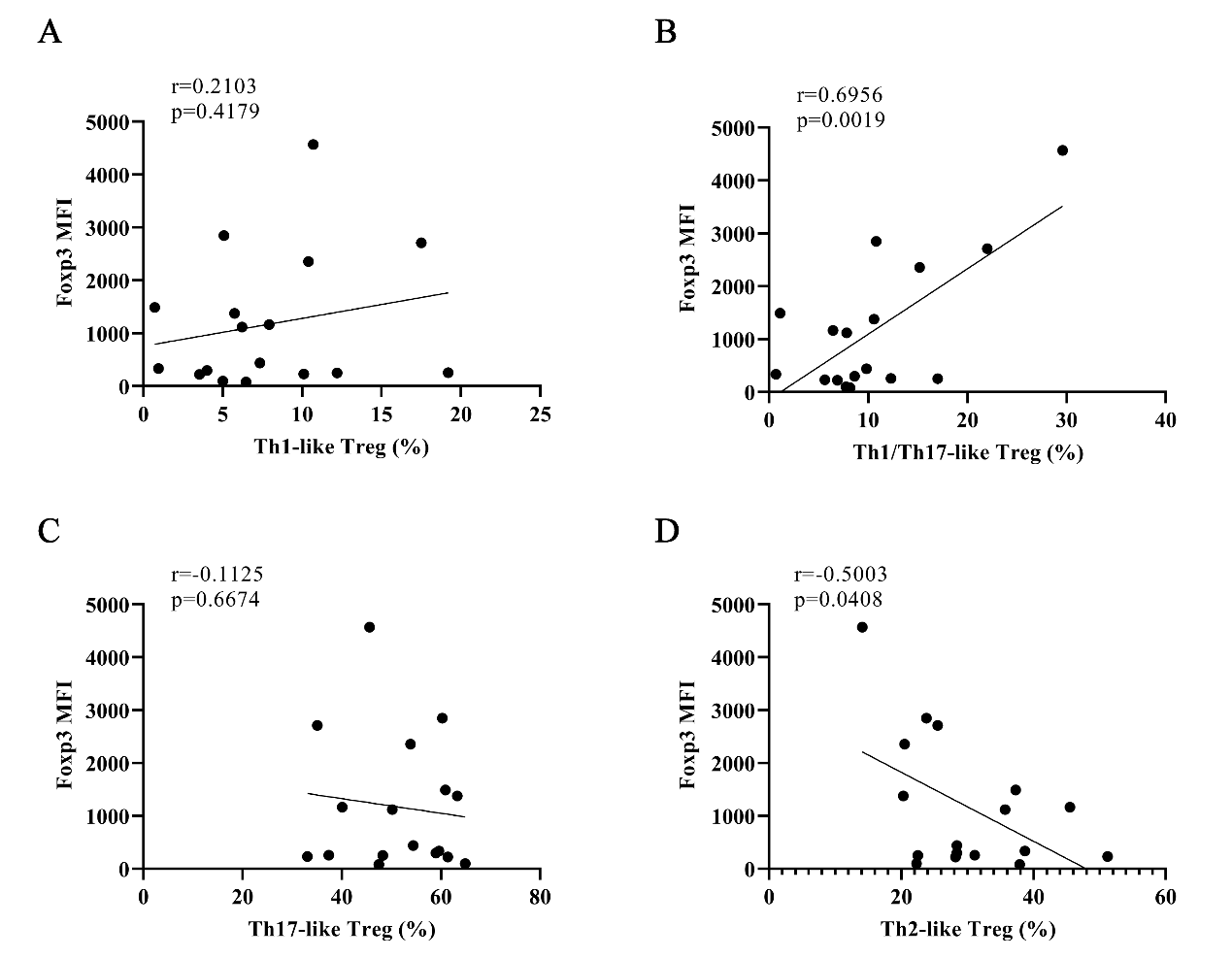


**Fig S6.** The correlation analysis between the expression of Foxp3 and circulating Th-like Treg subgroups. (A), (B), (C), (D) represent the Th1-like, Th1/Th17-like, Th17-like, and Th2-like phenotype, respectively.


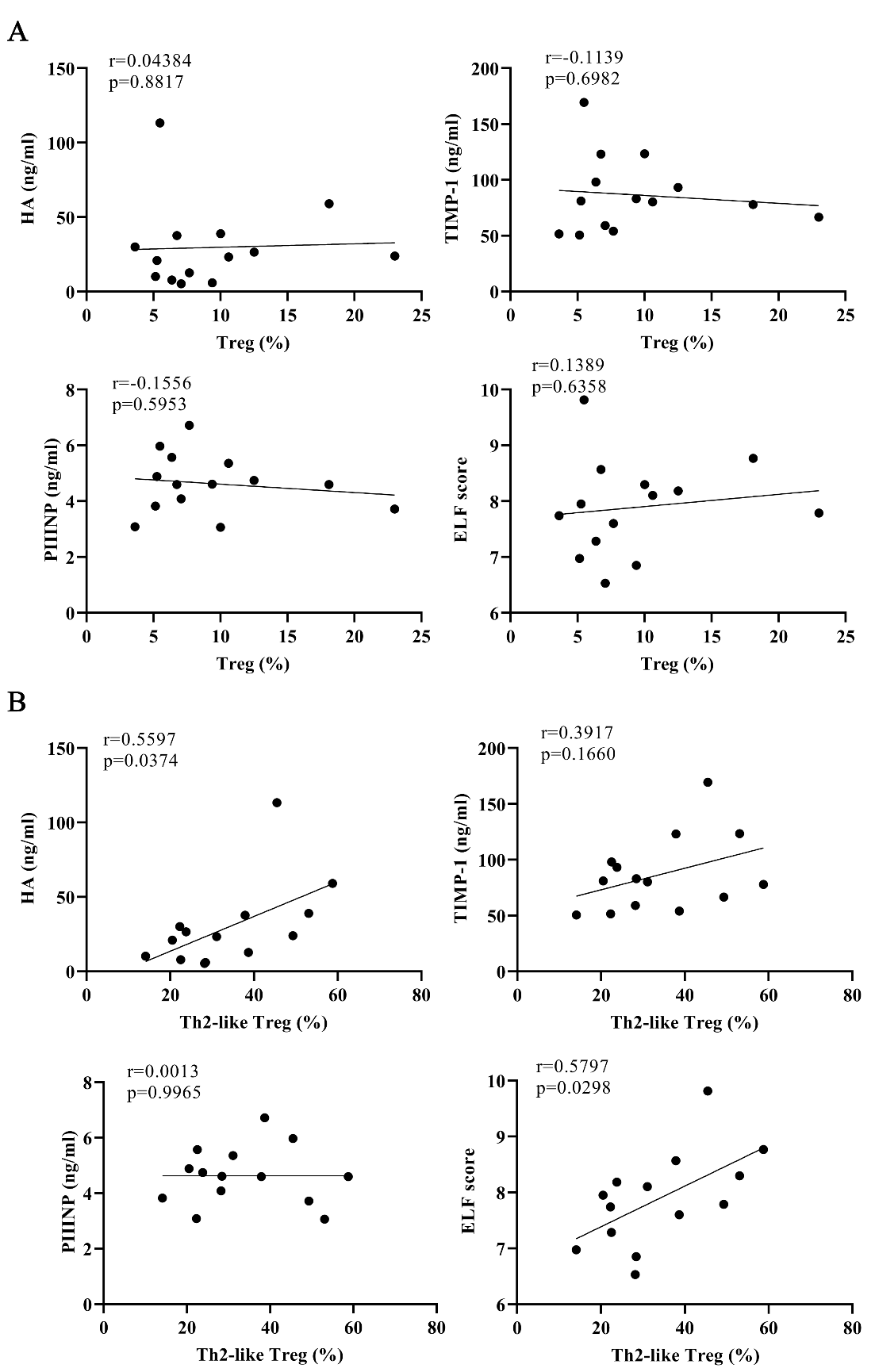


**Fig S7.** The correlation analysis between circulating Th-like Treg subsets and plasma fibrosis marker levels. (A) The correlation analysis between circulating Tregs and plasma fibrosis marker levels. (B) The correlation analysis between Th-like Tregs and plasma fibrosis marker levels.


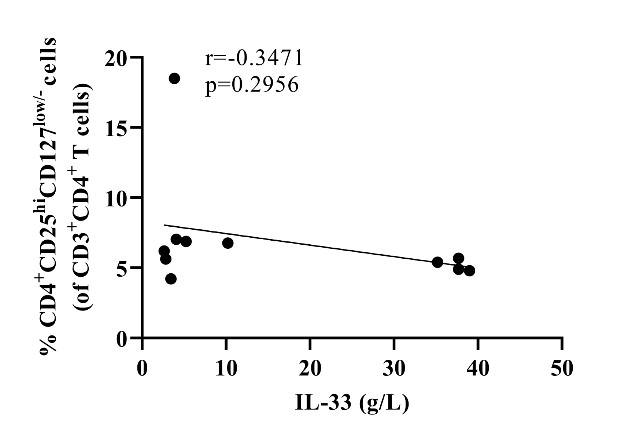


**Fig S8.** The correlation between circulating Tregs and plasma IL-33.


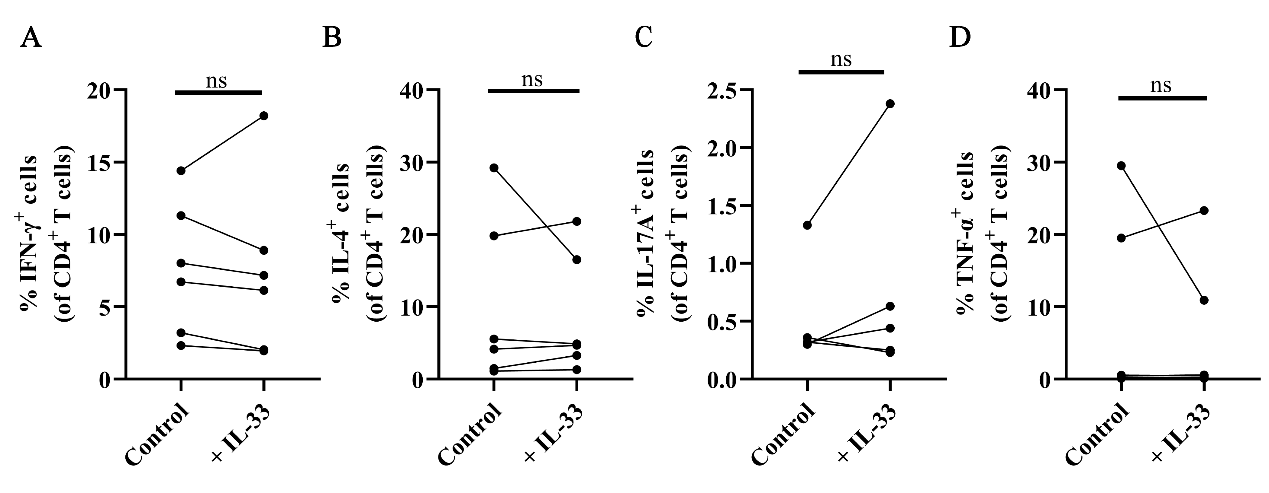


**Fig S9.** Interferon (IFN)-γ (A), IL-4 (B), interleukin (IL)-17A (C) and Tumor necrosis factor (TNF)-α (D) secreted by FACS-sorted Tconvs from IOI patients were detected after stimulation with IL-33 in the presence of anti-CD3 and anti-CD28 and IL-2. ns, not significant.


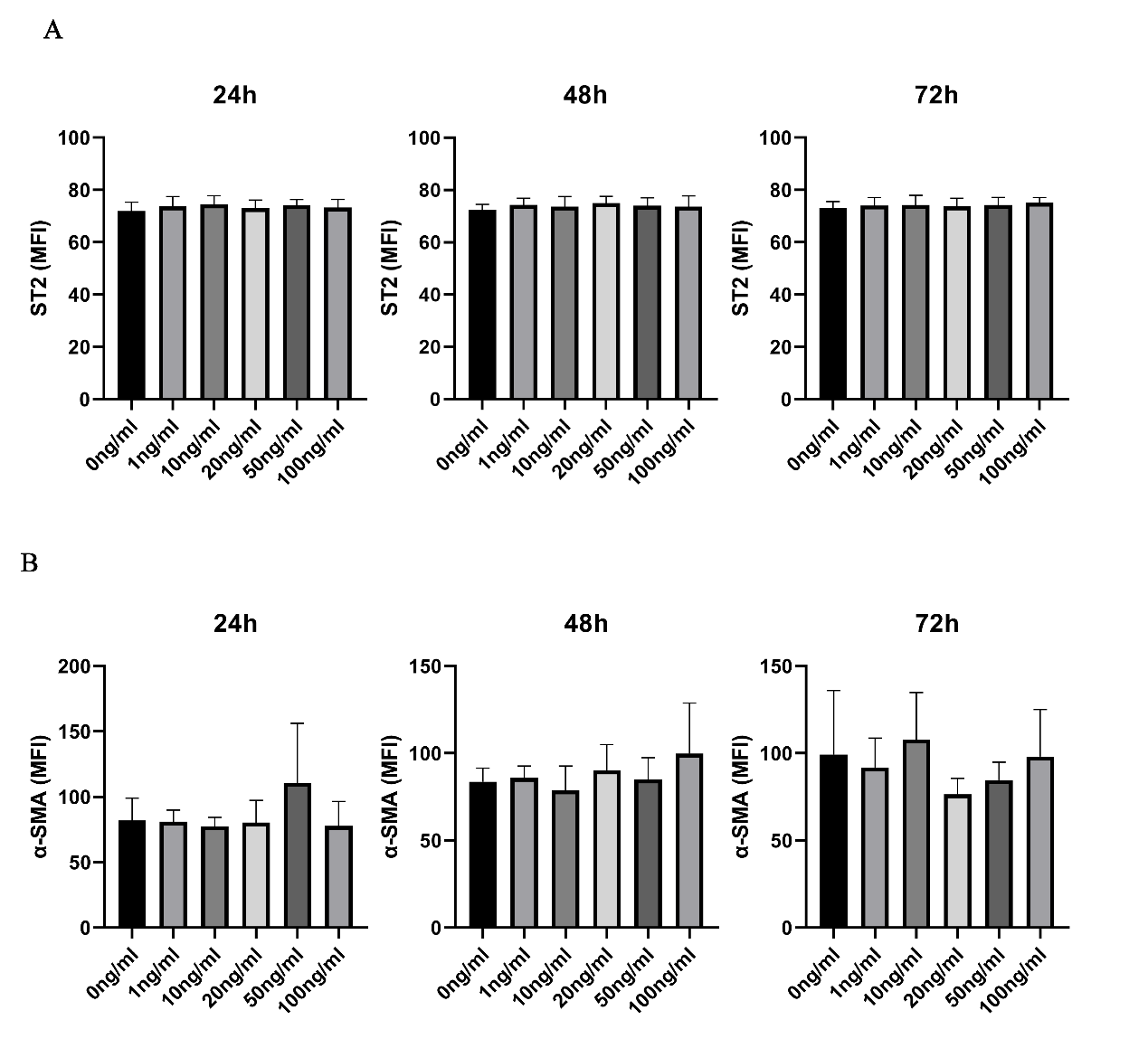


**Fig S10.** IL-33 stimulated OFs with different concentrations. (A) The expression of ST2 in OFs after stimulation. (B) The expression of α-SMA in OFs after stimulation. ns, not significant.
